# Artificial Intelligence Driven Biomedical Image Classification for Robust Rheumatoid Arthritis Classification

**DOI:** 10.3390/biomedicines10112714

**Published:** 2022-10-26

**Authors:** Marwa Obayya, Mohammad Alamgeer, Jaber S. Alzahrani, Rana Alabdan, Fahd N. Al-Wesabi, Abdullah Mohamed, Mohamed Ibrahim Alsaid Hassan

**Affiliations:** 1 Department of Biomedical Engineering, College of Engineering, Princess Nourah bint Abdulrahman University, P.O. Box 84428, Riyadh 11671, Saudi Arabia; 2 Department of Information Systems, College of Science & Art at Mahayil, King Khalid University, Abha 62217, Saudi Arabia; 3 Department of Industrial Engineering, College of Engineering at Alqunfudah, Umm Al-Qura University, Mecca 92254, Saudi Arabia; 4 Department of Information Systems, College of Computer and Information Science, Majmaah University, Al-Majmaah 11952, Saudi Arabia; 5 Department of Computer Science, College of Science & Art at Mahayil, King Khalid University, Abha 62217, Saudi Arabia; 6 Research Centre, Future University in Egypt, New Cairo 11845, Egypt; 7 Department of Computer and Self Development, Preparatory Year Deanship, Prince Sattam bin Abdulaziz University, AlKharj 16242, Saudi Arabia

**Keywords:** biomedical images, artificial intelligence, wearables, rheumatoid arthritis, sensors, deep learning, medical data classification

## Abstract

Recently, artificial intelligence (AI) including machine learning (ML) and deep learning (DL) models has been commonly employed for the automated disease diagnosis process. AI in biological and biomedical imaging is an emerging area and will be a future trend in the field. At the same time, biomedical images can be used for the classification of Rheumatoid arthritis (RA) diseases. RA is an autoimmune illness that affects the musculoskeletal system causing systemic, inflammatory and chronic effects. The disease frequently becomes progressive and decreases physical function, causing articular damage, suffering, and fatigue. After a time, RA causes harm to the cartilage of the joints and bones, weakens the tendons and joints, and finally causes joint destruction. Sensors (thermal infrared camera sensor, accelerometers and wearable sensors) are more commonly employed to collect data for RA. This study develops an Automated Rheumatoid Arthritis Classification using an Arithmetic Optimization Algorithm with Deep Learning (ARAC-AOADL) model. The goal of the presented ARAC-AOADL technique lies in the classification of health disorders depending upon RA and orthopaedics. Primarily, the presented ARAC-AOADL technique pre-processes the input images by median filtering (MF) technique. Then, the ARAC-AOADL technique uses AOA with an enhanced capsule network (ECN) model to produce feature vectors. For RA classification, the ARAC-AOADL technique uses a multi-kernel extreme learning machine (MKELM) model. The experimental result analysis of the ARAC-AOADL technique on a benchmark dataset reported a maximum accuracy of 98.57%. Therefore, the ARAC-AOADL technique can be employed for accurate and timely RA classification.

## 1. Introduction

Biomedical imaging acts as a vital part in the domain of biology and biomedicine, offering data related to the structural and functional mechanism of cells and the human body. Biological and biomedical imaging comprises microscopy, molecular imaging, pathological imaging, optical coherence tomography, nuclear medicine, ultrasound imaging, X-ray radiography, computed tomography, magnetic resonance imaging, and so on. The typical medical phenotype is labelled as non-infectious persistent polyarticular swelling, specifically of small joints, that causes progressive joint destruction and deformity and bone erosion, i.e., rheumatoid arthritis (RA) [1]. However, persistent periarticular synovitis, regardless of the immunopathogenesis, was linked to joint erosion and destruction, so regardless of the immune trigger a similar medical phenotype arises [2]. A logical extension of this view is that clinically different cases are linked with the medical RA phenotype. An unfulfilled necessity occurs in the translational setting for developing a robust pattern for assessing, diagnosing and prognosing patients affected with polyarthritis characteristic of early RA, particularly in recent times where the key role of autoinflammation or innate immunity is effectively detected in other chronic inflammatory illnesses [3]. Here, a new classification is proposed for the full medical disease spectrum of RA by utilizing the paradigmatic shift that occurred, in addition to the description of autoimmunity against citrullinated antigens in numerous RA cases. This immunological disease continuum method of inflammation in RA has consequences for therapeutic techniques [4,5].

Particularly viable reasons for applications utilizing the ensemble ML technique include hospital-based applications, controlling smart homes, information on-request systems, monitoring systems, outpatient care and mobile games communication interface [6]. Additionally, machine learning (ML) can be used in the development and evaluation of electroencephalogram (EEG)-related brain activities to measure using a biosensor [7]. With the developments in on-body wearable sensors and sensor technology, the ML technique will perform effectively in RA disease categorization. The author used a wearable glove-related sensor IoT for identifying RA diseases. Certainly, thermal structure-related camera sensors can be used to monitor temperature disparities in finger joints in several analyses [8]. ML includes different algorithms, procedures and techniques for finding limited associations within particular data and to produce tools constituting prescription, prediction or description combinations. ML has been initiated with many clinical domains and has been exemplified as very accurate in classifying and identifying different illnesses [9]. ML is a commonly used method for enhancing medical services and disease diagnosis with medical data development in various medical fields. Studies of effective applications of artificial intelligence (AI), which includes deep learning (DL) and ML methods. have seen an exponential growth in healthcare and medical fields [10]. Such techniques are critical in offering high-quality care to patients with RA. 

This study develops an Automated Rheumatoid Arthritis Classification using an Arithmetic Optimization Algorithm with Deep Learning (ARAC-AOADL) model. The goal of the presented ARAC-AOADL technique lies in the classification of health disorders depending upon RA and orthopaedics. Primarily, the presented ARAC-AOADL technique pre-processes the input images by median filtering (MF) technique. Then, the ARAC-AOADL technique uses AOA with an enhanced capsule network (ECN) model to produce feature vectors. For RA classification, the ARAC-AOADL technique uses a multi-kernel extreme learning machine (MKELM) model. The experimental results analysis of the ARAC-AOADL technique is tested on two medical datasets and the results are examined under several aspects.

## 2. Related Works

Lim et al. [11] modelled a new feature engineering technique compiling potentially functional coding haplotypes (pfcHap), including ML feature selection to detect biologically meaningful, probably causative genetic factors, that considers effective SNP–SNP interactions in the pfcHap to optimally forecast the methotrexate (MTX) response in RA patients. Ahalya et al. [12], by utilizing modified pre-trained CNN techniques, produced automated patch-related classification of hand Xray images, and then for for automated classification and feature extraction of hand Xray images and, for comparing the efficiency of CNN techniques with linear and non-linear kernels, a customized CNN technique was developed; they finally classified the normal and RA by employing ML methods and framing the hand-crafted feature fusion (SIFT and Customized CNN features).

Yang et al. [13] introduced a grading technique to estimate and detect the texture and geometric features of bone erosion and synovium thickening. This study will use the metrics and texture features of ROI in a dissimilar way to previous studies in this area. The segmented outcomes were examined for the extraction of three quantitative geometric variables, which are integrated with GLCM statistic texture features to describe the ultrasonic image of metacarpophalangeal RA. Tang et al. [14] modelled an automated RA grading technique leveraging DCNN for assistance in medical assessment. Here, the input is the Gray-scale ultrasound images of finger joints whereas the output is the RA grading outcomes. The authors executed data augmentation for increasing the training samples count. The authors pre-trained the GoogLeNet on ImageNet as an extracting feature and then fine-tuned them.

In [15], the classification of clinical disorders related to RA and orthopaedics dataset utilizing Ensemble techniques was conferred. The RA data was collected from the study of WBC classification by utilizing features derived from the lymphocyte image obtained through a digital microscope. The orthopaedic datasets are a benchmark dataset for this work, since they imposed the same classifier issue with some numerical features. In this study, three ensemble techniques, random subspace, bagging and Adaboost, were used. Such ensemble techniques use RF and kNN as the base learners of the ensemble techniques. In [16], for enhancement of disease risk evaluation, ML and matrix factorization methods were combined to find significant and implicit risk factors. A new structure was modelled that can successfully evaluate early disease risks and RA was employed as a case study. This structure has three main phases: 1early disease risk assessment, data preprocessing, and risk factor optimization. This was the first study compiling ML and matrix factorization for disease risk evaluation implemented with nation-wide and longitudinal medical diagnostic databases. Andreu-Perez et al. [17] formulated a technique that could produce optimally grained actigraphies for capturing the effect of the disease on the daily actions of patients. A study of processing techniques related to ML and DL was offered.

## 3. The Proposed Model

**Figure 1:**
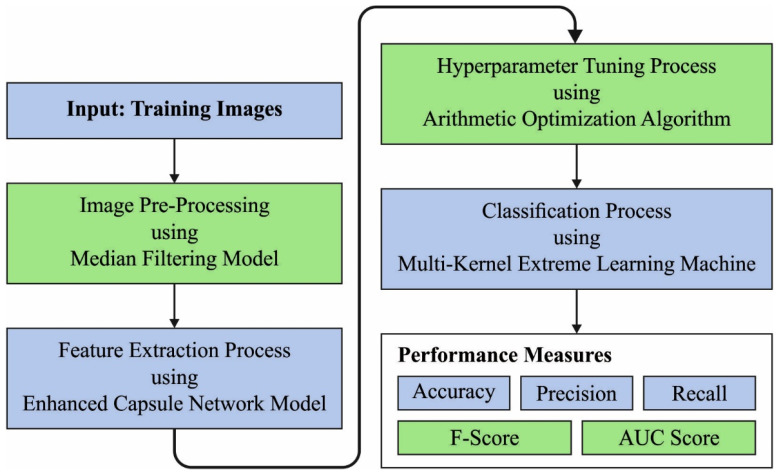
Working Process of ARAC-AOADL technique.

In this study, we have developed a new ARAC-AOADL technique for accurate RA classification, which helps to identify the health disorders depending upon RA and orthopaedics. The presented ARAC-AOADL technique encompasses MF-based noise removal, ECN feature extraction, AOA hyperparameter tuning and MKELM classification. The working of the 1ARAC-AOADL technique is depicted in Figure 1.

### 3.1. Noise Filtering Technique

Primarily, the presented ARAC-AOADL technique pre-processes the input images by the MF technique. Initially, the input image was preprocessed by means of the MF algorithm for getting rid of the noise within them. MF based on specificity is one of the applications from medical image noise extraction. The major concept behindhand MF is to present a process for assembling each neighborhood from the increasing order, which selects the median values of arranged numbers and replaces the central pixel as follows:

(1) $$y_{\left(i,\;j\right)}=median\left\{x_{\left(i,j\right)},\;\left(i,\;j\right)\in C\right\},\;$$

where C indicates the central neighborhood location of the image. In such cases, the MF was executed by digital noise extraction from the input images, whereby the filter mask with size 3×3 was applied.

### 3.2. Feature Extraction Using Optimal ECN Model

In this study, the ARAC-AOADL technique uses AOA with the ECN models to produce the feature vector. In the presented technique, the splitted pixel set of the images can be labelled as a collection of nerve cells corresponding to the capsule [18]. Consider $Y_{i}\in$ [healthy, tumor] as i-th output capsules, and $we_{ij}$ signifies the weighted matrices in the following:

(2) $${\hat{y}}_{\left(ij\right)}=we_{ij}y_{ij}$$

In Equation (5), ${\hat{y}}_{\left(ij\right)}$ describes the detection vector that diagnoses output parent j-th capsules with i-th capsule, and pixel range was applied to evaluate the weight quantity. The quantity for the weight was improved if the value was decreased or the pixel involves the positive group. The softmax method is exploited by the preceding layer capsule and the potential parent capsule as a coefficient is encoded $c_{ij}$ wherein major logits $b_{ij}$ show the log preceding probability of i-th routing capsules in the preceding layer to j-th capsules in the succeeding layer. Generally, the “routing-by-agreement” methodology was executed by logit for the capsule in all the layers:

(3) $$c_{ij}=\frac{e^{b_{ij}}}{\sum_{i}e^{b_{ij}}}$$

The previous layer demonstrates key elements to compute the input of j-th parent capsules as follows:

(4) $$s_{j}=\sum_{i}c_{ij}{\hat{y}}_{\left(i|j\right)}$$

The compressed pixel vector can be defined in (0, 1) by a non-linear method called squashing and it is computed by the following expression:

(5) $$va_{j}=\frac{\|s_{j}|{|}^{2}}{1+\|s_{j}{\|}^{2}}\times \frac{s_{j}}{\varepsilon +||s_{j}{\|}^{2}}$$

where $\varepsilon =10^{-7}$. The subsequent layer capsule was attained using:

(6) $$a_{ij}=va_{j}\times {\hat{y}}_{\left(ij\right)}$$

The entire capsule classifier is regarded as margin loss $\left(Loss_{k}\right)$ in the class capsule k for capsule network based on the loss:

(7) $$Loss_{k}=T_{k}\;\max\;{\left(0,\;m^{+}-\|va_{k}\|\right)}^{2}+\lambda \left(1-T_{k}\right)\;\max\;{\left(0,\;\|va_{k}\|-m^{-}\right)}^{2}$$

For the hyperparameter selection process, the AOA is exploited. AOA mainly replicates the use of the arithmetical operator during the arithmetical problem-solving method. Arithmetic is the part of mathematics that is exploited to handle the property of operation and numbers. An arithmetical operator is an operation symbol that executes fundamental arithmetic, i.e., a symbol applied to four processes. During optimization, these operators are utilized for selecting the best solution from the candidates. The optimization technique is based on two main processes: exploration and development. Initially, the search space of candidate solution is expansively protected for breaking the deadlock of the methodology within the search stagnation. Then, the performance for the solution is searched more deeply.

The series of possible solutions was arbitrarily generated in the initial stage of the optimization method of AOA, as given below.

(8) $$X=\left[\begin{array}{cccccc} x_{1,1} & \ldots & \ldots & x_{1,j} & x_{1,n-1} & x_{1,n}\\ x_{2,1} & \ldots & \ldots & x_{2,j} & \ldots & x_{2,n}\\ \ldots & \ldots & \ldots & \ldots & \ldots & \ldots \\ \vdots & \vdots & \vdots & \vdots & \vdots & \vdots \\ x_{N-1,\;1} & \ldots & \ldots & x_{N-1,j} & \ldots & x_{N-1,n\;}\\ x_{N,1} & \ldots & \ldots & x_{N,j} & x_{N,n-1} & x_{N,n} \end{array}\right]\;\;\;\;\;\;\;$$

Formerly, the AOA implements the optimized methodology, necessitating the resolution of the search process in accordance with the value of the Math Optimizer Accelerated (MOA) process that is calculated by the following equation.

(9) $$MOA\left(C\_Iter\right)=\mathrm{Min}+C\_Iter\times \left(\frac{\mathrm{Max}-\;\mathrm{Min}\;}{M_{-}Iter}\right)$$

In Equation (9), $MOA\left(C\_Iter\right)$ indicates the function value in C_Iter iteration; C_Iter represent the present iteration; M_Iter denotes the maximal iterations count; Min and Max are accelerated function minimal and maximal values.

The exploration phase in the AOA method is realized generally by Division (D) and Multiplication (M) operators [19]. During mathematical computation, these two operators accomplish distributed value for a wide-ranging coverage of candidate solutions. The location of candidate solution is upgraded considerably in the exploration procedure, as follows:

(10) $$x_{i,j}\left(C\_Iter+1\right)=\left\{\begin{array}{ll} best\left(x_{j}\right)/\left(MOP+\varepsilon \right)\times \left(\left(UB_{j}-LB_{j}\right)\times \mu +LB_{j}\right) & r_{2}<0.5\\ best\left(x_{j}\right)\times MOP\times \left(UB_{j}-LB_{j}\right)\times \mu +LB_{j} & otherwise \end{array}\right.$$

In Equation (16), $\chi_{i,j}\left(C\_Iter+1\right)$ denotes the *j*th location of *i*th solution in $\left(C\_Iter+1\right)\mathrm{th}$ iteration; ε indicates the small values; UB and LB indicates the upper and lower bounds of the location of candidate solution; μ is employed for regulating the exploration stage set to 0.5; the MOP signifies the math optimizer probability of AOA that is described in the following:

(11) $$MOP\;\left(C\_Iter\right)=1-\frac{C_{-}Iter^{\frac{1}{\alpha }}}{M_{-}Iter^{\frac{1}{\alpha }}}$$

Now, α defines the accuracy of exploitation on the iteration, α=5. The execution of exploitation method depends mainly on Subtraction (S) and Addition (A) operators that are easier to cause minimal dispersion, for the candidate solution is executed by a deep searching with larger probability of estimating the optimal solution [20]. In the growth step, the candidate solution was upgraded as follows:

(12) $$x_{i,j}\left(C_{Iter}+1\right)=\left\{\begin{array}{ll} best\left(x_{j}\right)-MOP\times (\left(UB_{j}-LB_{j}\right)\times \mu +LB_{j}, & r_{3}<05\\ best\left(x_{j}\right)+M0P\times (\left(UB_{j}-LB_{j}\right)\times \mu +LB_{j}, & otherwise \end{array}\right\}$$

The adaptive conversions among the exploration and exploitation stages are supported by the AOA method which defines an optimal solution and continues with a diversity of possible solutions to conduct a wide-ranging search as illusetrated in Algorithm 1.
**Algorithm 1.** Pseudocode of AOAInitialization of the parameter pop-size (N) and maximal iteration (T)Initialization of the location of every search agent $X_{i}\left(i=1,2,\;\ldots ,\;N\right)$
Set the parameters
Min and Max
While $\left(t\leq T\right)$
    Evaluate the fitness of all the search agent Upgrade bestFitness, $X_{b}$
    Evaluate the MOP    Evaluate the MOA    For every search agent        If rand>MOA            Upgrade position        Else            Upgrade position        End if    End for                   t=t+1End WhileReturn best Fitness, $X_{b}$

### 3.3. RA Classification Model

**Figure 2:**
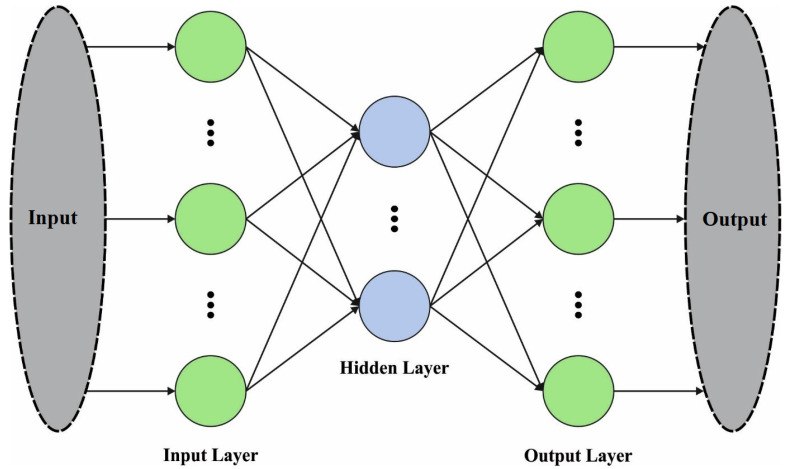
Structure of ELM model.

For RA classification, the ARAC-AOADL technique employed the MKELM model. The standard KELM is a single kernel based model. The structure of standard ELM is shown in Figure 2. Since a distinct kernel function provided a similar measure to the sample point, the efficacy of the kernel function could be based considerably to the related dataset. The input signal has features of considerable amount, irregular distribution of instances generated using imbalance, and maximal dimension feature space. Utilizing a single kernel to process the input dataset could not solve the problems effectively. The kernel function was regarded as global or local kernel function depending on rotation or translation invariances [21]. The global kernel function was higher at removing global features, and the local kernel function was better at eliminating the local feature of instance. In multi-kernel learning, an optimal kernel was regarded as linear integration of the group of base kernels, and the better linear integration coefficient and the classification parameter are learned equally using the margin maximization. The polynomial kernel and RBF are global and local kernel functions with optimal efficacy.

In order to balance the integration of the classifier efficiency and generalized capability, an MKELM has been generated using linear integration of the polynomial kernels and RBF and they are defined in the following equation:

(13) $$K_{mix}=\lambda k_{rbf}\left(u,u_{i}\right)+\left(1-\lambda \right)k_{poly}\left(u,u_{j}\right))$$

where λ(0<λ<1) denotes the weighted coefficient of linear integration.

(14) $$k_{rbf}\left(u,u_{j}\right)=\exp\left(\frac{{-||u-u_{i}||}^{2}}{2\sigma^{2}}\right),$$

(15) $$k_{poly}\left(u,u_{i}\right)={\left(u\cdot u_{i}+1\right)}^{d},$$

From the expression, d indicates fixed to two, as the dimension of polynomial space refers to $n^{d}$; once the sample size corresponded to thousands and the index corresponded to three, the dimension can be accomplished as 1 billion, and the computation of the inner product generates a dimension disaster [22].

Eventually, the resulting objective of MKELM was defined by the following expression:

(16) $$f\left(x\right)=\left[K_{mix}\left(u,u_{1}\right)\ldots K_{mix}\left(u,u_{N}\right)\right]{\left(\frac{1}{C}+M\right)}^{-1}\;T.$$

## 4. Experimental Validation

The proposed model is simulated using Python 3.6.5 tool on PC i5-8600k, GeForce 1050Ti 4GB, 16GB RAM, 250GB SSD, and 1TB HDD. The parameter settings are given as follows: learning rate: 0.01, dropout: 0.5, batch size: 5, epoch count: 50, and activation: ReLU. The experimental validation of the ARAC-AOADL model is performed on a benchmark dataset from the Kaggle repository [23]. The dataset holds 310 samples with three classes, as given in Table 1.

**Figure 3:**
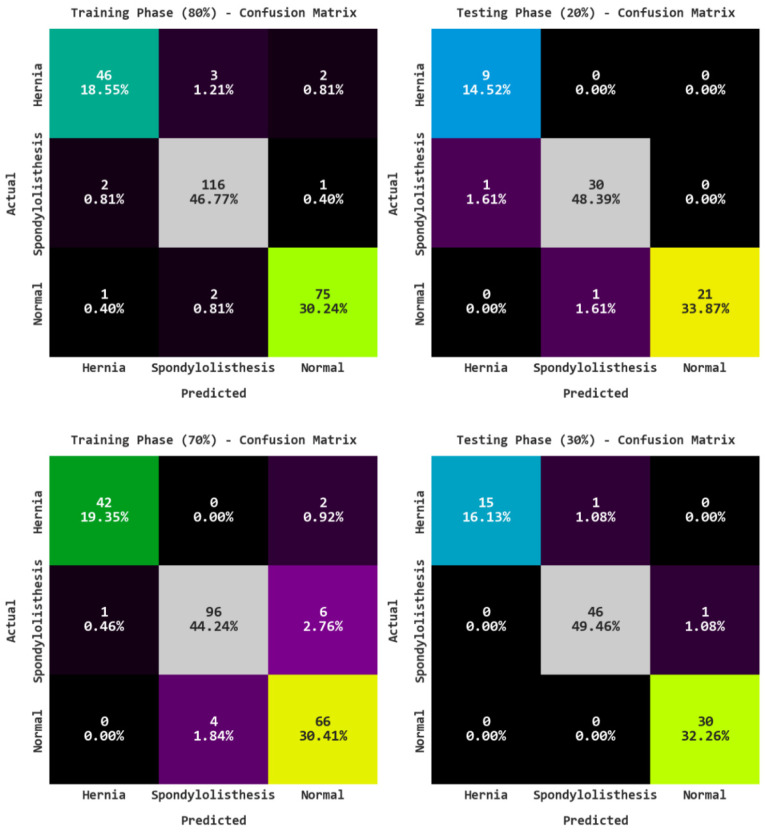
Confusion matrices of ARAC-AOADL model.

Figure 3 shows the confusion matrices of the ARAC-AOADL model on various training (TR) and testing (TS) data. The figure highlights that the ARAC-AOADL model has reached effective RA classification results.

Table 2 offers a detailed classifier results of the ARAC-AOADL model on 80% of TR data and 20% of TS data. The results reveal the RA classification results of the ARAC-AOADL model on 80% of TR data. The ARAC-AOADL model has recognized Hernia class samples with $accu_{y}$ of 96.77%, $prec_{n}$ of 93.88%, $reca_{l}$ of 90.20%, $F_{score}$ of 92%, and $AUC_{score}$ of 94.34%. In addition, the ARAC-AOADL model has categorized Normal class samples with $accu_{y}$ of 97.58%, $prec_{n}$ of 96.15%, $reca_{l}$ of 96.15%, $F_{score}$ of 96.15%, and $AUC_{score}$ of 97.19%. Moreover, the ARAC-AOADL model has reached average $accu_{y}$ of 97.04%, $prec_{n}$ of 95.30%, $reca_{l}$ of 94.61%, $F_{score}$ of 94.94%, and $AUC_{score}$ of 96.11%. The ARAC-AOADL method has recognized Hernia class samples with $accu_{y}$ of 98.39%, $prec_{n}$ of 90%, $reca_{l}$ of 100.00%, $F_{score}$ of 94.74%, and $AUC_{score}$ of 99.06%. Furthermore, the ARAC-AOADL technique has categorized Normal class samples with $accu_{y}$ of 98.39%, $prec_{n}$ of 100%, $reca_{l}$ of 95.45%, $F_{score}$ of 97.67%, and $AUC_{score}$ of 97.73%. In addition, the ARAC-AOADL approach has reached average $accu_{y}$ of 97.85%, $prec_{n}$ of 95.59%, $reca_{l}$ of 97.41%, $F_{score}$ of 96.40%, and $AUC_{score}$ of 97.85%.

**Figure 4:**
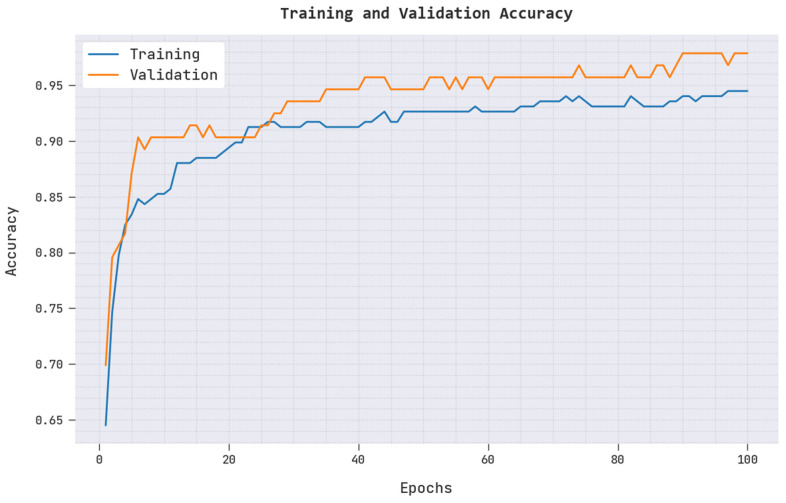
TRAC and VAAC of ARAC-AOADL model.

Table 3 shows the overall RA classification results on 70% of TR data and 30% of TS data. Figure 4 demonstrates the RA classification outcomes of the ARAC-AOADL technique on 70% of TR data. The ARAC-AOADL system has recognized Hernia class samples with $accu_{y}$ of 98.62%, $prec_{n}$ of 97.67%, $reca_{l}$ of 95.45%, $F_{score}$ of 96.55%, and $AUC_{score}$ of 97.44%. Furthermore, the ARAC-AOADL method has categorized Normal class samples with $accu_{y}$ of 94.47%, $prec_{n}$ of 89.19%, $reca_{l}$ of 94.29%, $F_{score}$ of 91.67%, and $AUC_{score}$ of 94.42%. Besides, the ARAC-AOADL technique has obtained average $accu_{y}$ of 96.01%, $prec_{n}$ of 94.29%, $reca_{l}$ of 94.31%, $F_{score}$ of 94.27%, and $AUC_{score}$ of 95.57%.

A brief training accuracy (TRAC) and validation accuracy (VAAC) of the ARAC-AOADL model is given in Figure 4. The results inferred that the ARAC-AOADL model has reached maximum TRAC and VAAC values. It is obvious that the VAAC is superior to TRAC.

**Figure 5:**
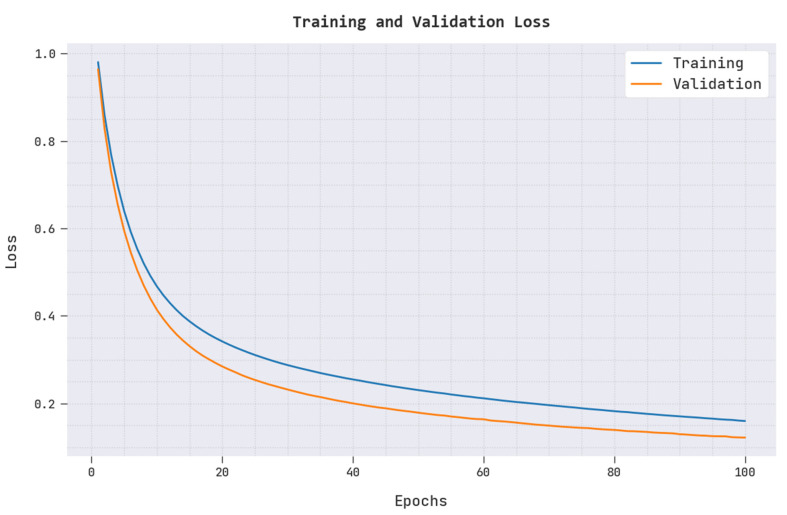
TRAL and VALL of ARAC-AOADL model.

In Figure 5, a clear training loss (TRAL) and validation loss (VALL) of the ARAC-AOADL model is reported. The figure reported that the ARAC-AOADL model has reached minimal values of TRAL and VALL.

**Figure 6:**
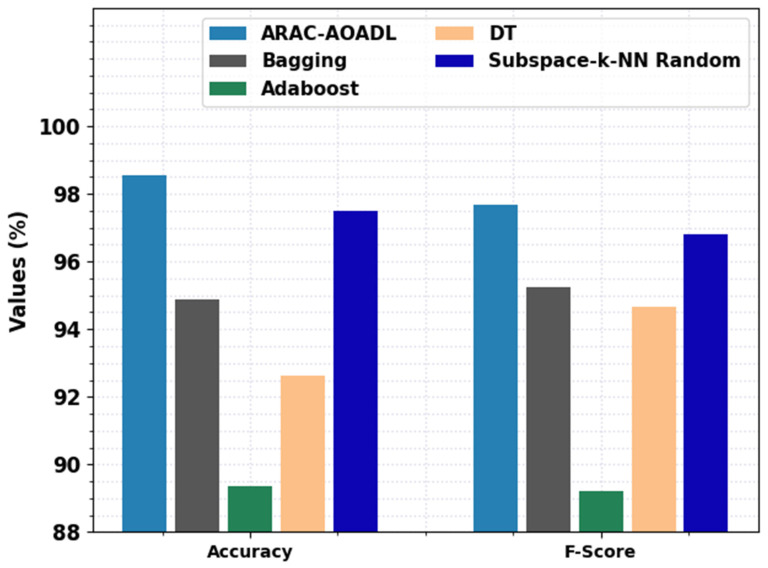
Comparative RA Classification results of ARAC-AOADL model interms of $accu_{y}$ and $F_{score}$.

To highlight the enhanced performance of the ARAC-AOADL model, a comparison study is made in Table 4 and Figure 6. An extensive comparison study of the presented ARAC-AOADL model with existing ML models in terms of $accu_{y}$ and $F_{score}$ is provided in Figure 6. The experimental values inferred that the ARAC-AOADL model has shown effective classification performance. For instance, based on $accu_{y}$, the ARAC-AOADL model has offered higher $accu_{y}$ of 98.57% whereas the Bagging, Adaboost, DT, and Subspace-k-NN random models have attained lower $accu_{y}$ of 94.89%, 89.37%, 92.64%, and 97.50% respectively. Furthermore, based on $F_{score}$, the ARAC-AOADL technique has provided maximum $accu_{y}$ of 97.67% while the Bagging, Adaboost, DT, and Subspace-k-NN random techniques have accomplished lower $accu_{y}$ of 95.23%, 89.21%, 94.68%, and 96.82%, correspondingly.

**Figure 7:**
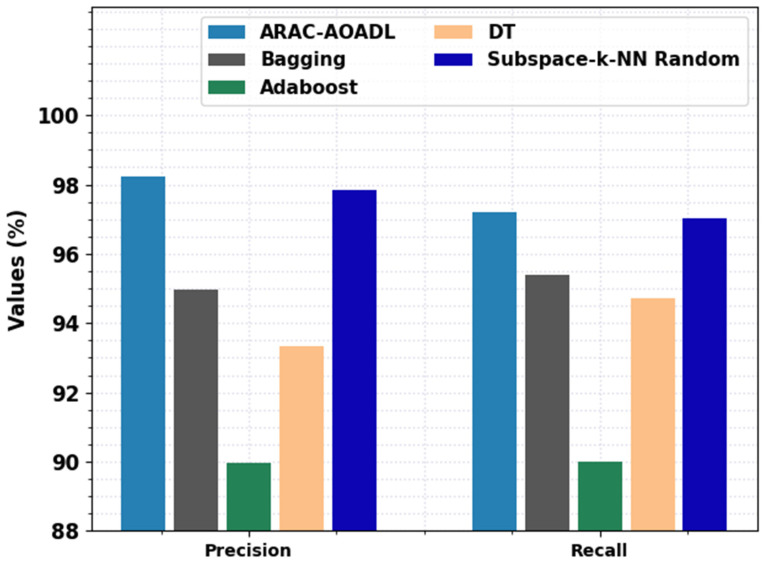
Comparative RA Classification results of ARAC-AOADL model interms of $prec_{n}$ and $reca_{l}$.

A comprehensive analysis of the proposed ARAC-AOADL methodology with current ML models with respect to $prec_{n}$ and $reca_{l}$ is given in Figure 7. The experimental value demonstrates that the ARAC-AOADL approach has demonstrated effective classification performance. For example, based on $prec_{n}$, the ARAC-AOADL technique has provided maximum $prec_{n}$ of 98.22% while the Bagging, Adaboost, DT, and Subspace-k-NN random models have accomplished lower $prec_{n}$ of 94.89%, 89.37%, 92.64%, and 97.50% correspondingly. Furthermore, based on $reca_{l}$, the ARAC-AOADL technique has given maximum $reca_{l}$ of 97.67% while the Bagging, Adaboost, DT, and Subspace-k-NN random approaches have accomplished lower $reca_{l}$ of 95.40%, 90.01%, 94.73%, and 97.02%, correspondingly.

After examining the detailed results, the proposed model has gained enhanced performance with maximum $accu_{y}$ of 98.57%, $prec_{n}$ of 98.22%, $reca_{l}$ of 97.21%, and $F_{score}$ of 97.67%. The enhanced performacne of the proposed model is due to the inclusion of the AOA based hyperparameter tuning process. Since the trial and error hyperparmaeter selection is not an effective process, the optimal hyperparmater tuning AOA helps to accomplish enhanced RA classification performance. Therefore, the proposed model can be employed for precise RA classification, which enables detection of health disorders based on RA and orthopaedics.

## 5. Conclusions

In this study, we have developed a new ARAC-AOADL technique for accurate RA classification, which helps to identify health disorders depending upon RA and orthopaedics. Primarily, the presented ARAC-AOADL technique pre-processes the input images by the MF technique. Then, the ARAC-AOADL technique uses AOA with the ECN model to produce feature vectors. For RA classification, the ARAC-AOADL technique employed the MKELM model. The experimental result analysis of the ARAC-AOADL technique is tested on two medical datasets and the results are inspected under several aspects. The simulation results ensured the enhancements of the ARAC-AOADL technique in terms of different measures. In future, we can extend the ARAC-AOADL technique by hybrid DL classification models.

## Figures and Tables

**Table 1:** Dataset used.

Class	No. of Instances
Hernia	60
Spondylolisthesis	150
Normal	100
**Total Number of Samples**	**310**

**Table 2:** RA Classification results of ARAC-AOADL model on 80:20 of TR/TS data.

Training/Testing (80:20)					
Labels	Accuracy	Precision	Recall	F-Score	AUC Score
**Training Phase**					
Hernia	96.77	93.88	90.20	92.00	94.34
Spondylolisthesis	96.77	95.87	97.48	96.67	96.80
Normal	97.58	96.15	96.15	96.15	97.19
**Average**	**97.04**	**95.30**	**94.61**	**94.94**	**96.11**
**Testing Phase**					
Hernia	98.39	90.00	100.00	94.74	99.06
Spondylolisthesis	96.77	96.77	96.77	96.77	96.77
Normal	98.39	100.00	95.45	97.67	97.73
**Average**	**97.85**	**95.59**	**97.41**	**96.40**	**97.85**

**Table 3:** RA Classification results of ARAC-AOADL model on 70:30 of TR/TS data.

Training/Testing (70:30)					
Labels	Accuracy	Precision	Recall	F-Score	AUC Score
**Training Phase**					
Hernia	98.62	97.67	95.45	96.55	97.44
Spondylolisthesis	94.93	96.00	93.20	94.58	94.85
Normal	94.47	89.19	94.29	91.67	94.42
**Average**	**96.01**	**94.29**	**94.31**	**94.27**	**95.57**
**Testing Phase**					
Hernia	98.92	100.00	93.75	96.77	96.88
Spondylolisthesis	97.85	97.87	97.87	97.87	97.85
Normal	98.92	96.77	100.00	98.36	99.21
**Average**	**98.57**	**98.22**	**97.21**	**97.67**	**97.98**

**Table 4:** Comparative RA Classification results of ARAC-AOADL model.

Methods	Accuracy	Precision	Recall	F-Score
ARAC-AOADL	98.57	98.22	97.21	97.67
Bagging	94.89	94.97	95.4	95.23
Adaboost	89.37	89.96	90.01	89.21
DT	92.64	93.32	94.73	94.68
Subspace-k-NN Random	97.50	97.83	97.02	96.82

## Data Availability

Data sharing not applicable to this article as no datasets were generated during the current study.
